# Aerosol Delivery With a Vibrating Mesh Nebulizer Across Tidal Volume-Based Pediatric Invasive Ventilation Models: An In Vitro Evaluation

**DOI:** 10.1097/CCE.0000000000001430

**Published:** 2026-06-04

**Authors:** Shinya Miura, Yoshihiro Igarashi, Kenta Shono, Saho Shima, Atsushi Kawaguchi

**Affiliations:** 1 Division of Pediatric Critical Care, Department of Pediatrics, St. Marianna University School of Medicine, Kawasaki, Japan.; 2 Department of Clinical Engineering, St. Marianna University Hospital, Kawasaki, Japan.

**Keywords:** aerosol therapy, mechanical ventilation, pediatric, tidal volume, vibrating mesh nebulizer

## Abstract

**CONTEXT:**

Despite the widespread use of vibrating mesh nebulizers (VMNs) for aerosol therapy during mechanical ventilation, there is limited pediatric evidence on how tidal volume (TV)-based ventilation models and commonly modified ventilator- and circuit-related factors affect aerosol delivery.

**HYPOTHESIS:**

We hypothesized that these ventilator- and circuit-related modifications would affect the efficacy of aerosol delivery with a VMN.

**METHODS AND MODELS:**

We conducted an in vitro experiment using six pediatric ventilation models with TVs set at 8 mL/kg (TV: 40–400 mL for 5–50 kg), representing children from infancy to adolescence. We nebulized salbutamol with a VMN positioned on the dry side of a heated humidifier, and quantified inhaled mass fraction by measuring the drug collected on a filter positioned distal to the endotracheal tube using a spectrophotometer. We evaluated the effects of TV-based models and variations in other settings, including endotracheal tube sizes, bias flows, inspiratory rise time, ventilator modes, circuit configurations, VMN positions, and drug concentrations.

**RESULTS:**

In the baseline model with a TV of 80 mL, the mean ± sd of inhaled mass fraction was 11.2% ± 0.3%. It increased progressively across the TV-based models: 3.5% ± 0.2% (TV: 40 mL), 7.9% ± 0.4% (TV: 56 mL), 15.7% ± 0.2% (TV: 120 mL), 19.4% ± 0.4% (TV: 160 mL), and 21.3% ± 1.7% (TV: 400 mL; all *p* < 0.001 vs. baseline). No significant differences were observed in variations of the other ventilatory, endotracheal tube-, or circuit-related settings.

**INTERPRETATION AND CONCLUSIONS:**

VMN aerosol delivery increased across TV-based ventilation models and was minimally affected by other ventilator settings, endotracheal tube size, and circuit-related modifications. Consistent aerosol delivery at a given TV suggests that TV is the dominant determinant of aerosol delivery efficacy.

**KEY POINTS**

**Question**: How do tidal volume-based pediatric ventilation models and commonly modified ventilator and circuit factors affect aerosol delivery with a vibrating mesh nebulizer during pediatric invasive mechanical ventilation?

**Findings**: This in vitro study examined six pediatric ventilation models with tidal volumes ranging from 40 to 400 mL. Aerosol delivery increased with higher tidal volumes and differed significantly across models. However, variations in other ventilator settings, endotracheal tube size, circuit configuration, nebulizer position, and drug dilution did not significantly impact aerosol delivery.

**Meaning**: Tidal volume is the primary determinant of aerosol delivery efficiency during pediatric invasive ventilation, while other commonly adjusted factors have minimal impact.

Aerosol therapy is commonly used for children with acute respiratory failure who require invasive mechanical ventilation (1, 2). However, delivering aerosols efficiently to mechanically ventilated patients remains challenging because drug delivery can be influenced by several factors, including nebulizer type, ventilator settings, humidification, circuit configuration, endotracheal tube size, and patient characteristics (1, 3–6).

Vibrating mesh nebulizers (VMNs) are widely used as they generally provide a higher delivery efficiency than the jet or ultrasonic nebulizers (3, 4). In vitro studies have shown that VMN performance may also vary depending on factors such as ventilator settings, circuit configuration, and nebulizer position. However, most of the evidence comes from adult ventilation models (1). When pediatric evaluations have been included, they have often simulated only one or two pediatric ventilation models, rather than multiple ventilation models representing a wide range of pediatric age groups (3, 7).

Importantly, developmental changes in lung size, airway dimensions, and ventilatory requirements from infancy to adolescence may also affect aerosol delivery efficacy (2, 8, 9). Particularly, tidal volume has been considered a key determinant of aerosol delivery efficacy (10, 11). However, the effects of age-representative, tidal volume-based pediatric ventilation models, as well as the effects of variations in commonly modified factors in clinical settings (e.g., other ventilatory parameters, endotracheal tube size, and circuit configuration), on aerosol delivery during pediatric invasive ventilation, have not been adequately studied (12).

Therefore, the aims of this study were to: 1) systematically evaluate the inhaled mass fraction of salbutamol delivered by a VMN across tidal volume-based pediatric invasive ventilation models and 2) assess the impact of commonly modified ventilator- and circuit-related factors within a controlled in vitro experimental setup.

## MATERIALS AND METHODS

### Study Design and Funding

This in vitro experiment was conducted at St. Marianna University School of Medicine and was funded by the Japan Society for the Promotion of Science (Grant-in-Aid for Early-Career Scientists, Grant No. 24K19510), which funded the costs of experimental materials and equipment. The funder had no role in the study design, conduct, analysis, interpretation, or reporting.

### Experimental Setup

**Figure 1.**
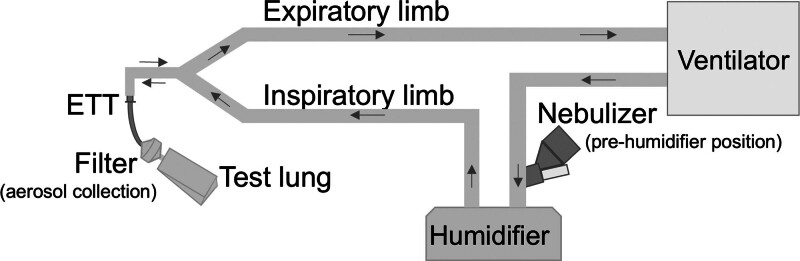
Experimental setup. An aerosol collection filter was positioned between the ETT and the test lung and sealed with paraffin film. *Arrows* indicate the direction of inspiratory and expiratory gas flow. ETT = endotracheal tube.

We assembled a pediatric lung model consisting of a mechanical ventilator (Puritan Bennett 980; Medtronic, Minneapolis, MN), a heated humidifier (MR290; Fisher & Paykel Healthcare, Auckland, New Zealand), and ventilator circuits selected according to ventilation models (RT266 and RT380, with inner diameters [IDs] of 10 and 22 mm, respectively, from Fisher & Paykel Healthcare). We positioned an aerosol collection filter between a size-appropriate endotracheal tube (Microcuff; Avanos Medical, Alpharetta, GA) and the test lung and sealed it with paraffin film (**Fig. 1**).

A VMN (Aerogen Solo; Aerogen, Galway, Ireland) was used for all experiments. The baseline ventilation model used pressure-regulated volume control ventilation with a tidal volume of 80 mL (8 mL/kg), respiratory rate of 25 breaths/min, inspiratory time of 0.7 seconds, positive end-expiratory pressure of 5 cm H_2_O, Fio_2_ of 0.21, and bias flow of 3.5 L/min, simulating a 10-kg 1-year-old child. The VMN was positioned on the dry side of the heated humidifier (pre-humidifier), as shown in Figure 1. The experimental room was maintained at 26°C with a relative humidity of 50%, and the circuit temperature was maintained at 38.0–38.5°C.

### Outcome and Aerosol Administration

The outcome was the inhaled mass fraction, defined as the ratio of the inhaled drug mass to the nominal dose placed in the nebulizer. A 2.5 mg dose of salbutamol (0.5%, 5 mg/mL) was diluted with normal saline to a total volume of 2.5 mL (five-fold dilution) and nebulized over around 10 minutes. Inhaled drug mass was quantified by measuring the amount of salbutamol captured on a filter positioned between the endotracheal tube and the test lung. After nebulization, the filter was eluted with ultrapure water, and the drug concentration was determined via ultraviolet-visible spectrophotometry at 276 nm (V-630; JASCO, Tokyo, Japan). Each condition was tested in triplicate, with additional runs performed if the variation exceeded 10%.

### Experimental Conditions

We systematically evaluated the following conditions (**Table 1**):

**TABLE 1.** Tidal Volume-Based Ventilation Models (Tidal Volume 8 mL/kg)

Tidal Volume, mL	Body Weight, kg	Respiratory Rate, Breaths/Min	Inspiratory Time, s	Positive End-Expiratory Pressure, cm H_2_O	Endotracheal Tube Inner Diameter, mm
40	5	30	0.6	5	3.0
56	7	30	0.6	5	3.0
80	10	25	0.7	5	3.5
120	15	20	0.75	5	4.0
160	20	18	0.9	5	4.5
400	50	15	1.0	5	7.5

These tidal volumes (40–400 mL) correspond to body weights of 5–50 kg, representing children from 2 mo to 15 yr old.

According to the institutional protocol, a ventilator circuit with an inner diameter of 10 mm was used for the 40-mL model, whereas circuits with an inner diameter of 22 mm were used for the other models.

- Tidal volume-based ventilation models: six models were defined by a tidal volume of 8 mL/kg (40–400 mL), corresponding to body weights of 5, 7, 10, 15, 20, and 50 kg (approximate ages: 2 mo to 15 yr), with a tidal volume of 80 mL used as the baseline ventilation model.
- Endotracheal tube size: Clinically plausible sizes with IDs ranging from 3.0 to 7.5 mm were selected based on the assumed body weight of the ventilation models. In a baseline ventilation model with tube modifications, the following IDs were compared: 3.0, 3.5, 4.0, and 4.5 mm.
- Bias flow: 2.0 and 3.5 L/min.
- Inspiratory rise time and ventilator mode: 20%, 50%, and 80% inspiratory rise time during pressure-regulated volume control ventilation, and constant inspiratory flow during volume-controlled ventilation.
- Circuit type: IDs of 10 and 22 mm, each with a length of 1.6 m.
- Circuit bends: straight, 180° bend, and 360° loop with a diameter of 30 cm.
- VMN position: pre-humidifier vs. post-humidifier.
- Drug dilution: undiluted, five-fold dilution, and 10-fold dilution.

We compared circuit types using the 56-mL model (7 kg) because both circuit sizes are used for patients of this body weight according to our institutional policy. Unless otherwise specified, all other experiments were conducted using the 80-mL baseline model.

### Statistical Analysis

The results were presented as the mean ± sd. All comparisons were performed using Student *t* test. All tests were two-sided, and a *p* value of less than 0.05 was considered statistically significant. The analyses were conducted using Stata 18 (StataCorp, College Station, TX).

## RESULTS

### Effect of the Tidal Volume-Based Ventilation Model

**Figure 2.**
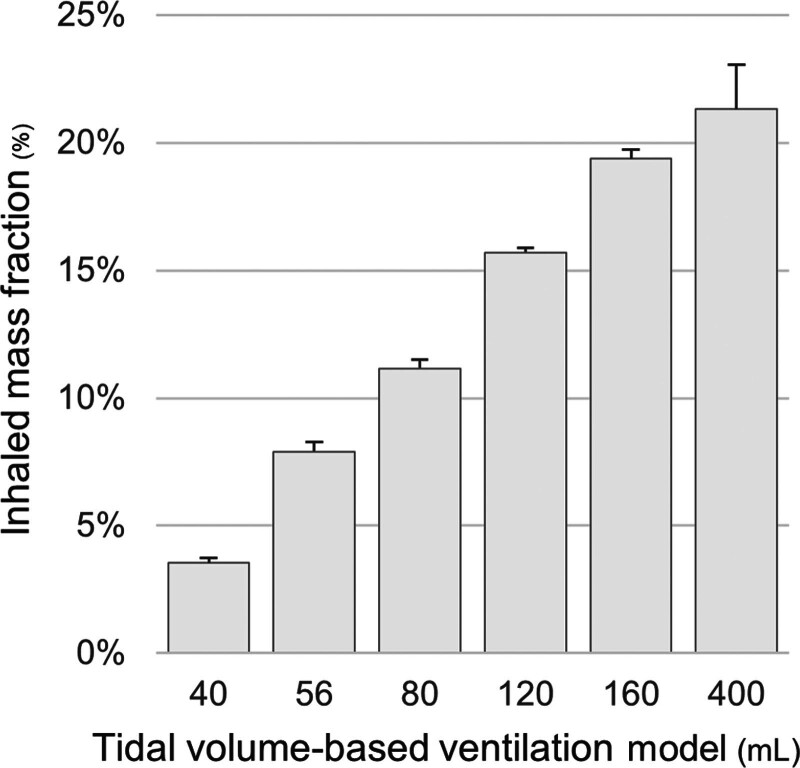
Inhaled mass fraction across tidal volume-based ventilation models. Inhaled mass fraction of salbutamol delivered by a vibrating mesh nebulizer across tidal volume-based pediatric ventilation models (tidal volume 8 mL/kg; 40–400 mL). Compared with the 80-mL baseline model, inhaled mass fraction differed significantly in all other models (all *p* < 0.001). *Bars* represent the mean values from triplicate experiments, and *error bars* indicate the sd.

In the 80-mL baseline model, inhaled mass fraction was 11.2% ± 0.3%. Inhaled mass fraction increased progressively across the tidal volume-based ventilation models, reaching 3.5% ± 0.2% at 40 mL, 7.9% ± 0.4% at 56 mL, 15.7% ± 0.2% at 120 mL, 19.4% ± 0.4% at 160 mL, and 21.3% ± 1.7% at 400 mL. Significant differences were observed between all tidal volume-based models and the 80-mL model (*p* < 0.001 for all comparisons; see **Fig. 2**).

### Effects of Ventilator Settings, Circuit Characteristics, and Nebulizer/Drug Conditions

#### Endotracheal Tube Size

**Figure 3.**
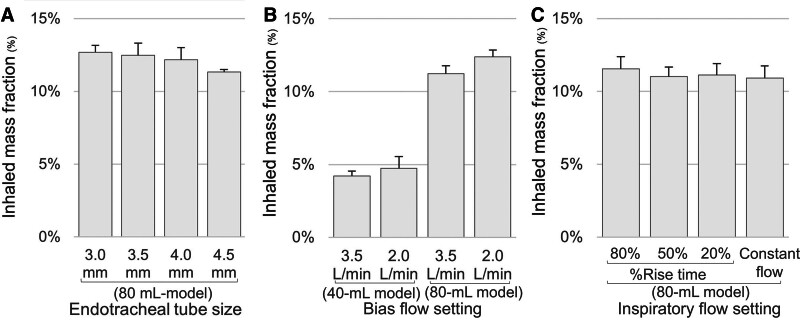
Inhaled mass fraction according to endotracheal tube size, bias flow, and inspiratory flow settings. Inhaled mass fraction was measured (**A**) using endotracheal tubes with inner diameters of 3.0, 3.5 (reference), 4.0, and 4.5 mm in the 80-mL baseline ventilation model; (**B**) under different bias flow settings in the 40- and 80-mL baseline ventilation models; and (**C**) under different inspiratory rise time settings (20%, 50%, and 80%) in pressure-regulated volume control ventilation, as well as in volume-controlled ventilation with a constant inspiratory flow, in the 80-mL baseline model. No significant differences were observed among the tested settings compared with the reference model. Although a slight decrease was observed with the 4.5 mm endotracheal tube, this was not statistically significant. *Bars* represent the mean values from triplicate experiments, and *error bars* indicate the sd.

Compared with the 80-mL baseline model using a 3.5-mm endotracheal tube, inhaled mass fraction did not differ significantly with the 3.0-mm (12.7% ± 0.5%; *p* = 0.730), 4.0-mm (12.2% ± 0.8%; *p* = 0.663), and 4.5-mm (11.3% ± 0.2%; *p* = 0.078) tubes (**Fig. 3**). Although a slight decrease was observed with the 4.5-mm tube, it was not statistically significant.

#### Bias Flow

Reducing the bias flow from 3.5 to 2.0 L/min did not significantly increase inhaled mass fraction in either the 40-mL model (4.2% ± 0.3% vs. 4.7% ± 0.8%; *p* = 0.173) or in the 80-mL baseline model (11.2% ± 0.5% vs. 12.4% ± 0.5%; *p* = 0.051; Fig. 3).

#### Inspiratory Rise Time and Ventilator Mode

Compared with the 80-mL baseline model with an inspiratory rise time of 50%, inhaled mass fraction did not differ with inspiratory rise times of 80% (11.6% ± 0.8%; *p* = 0.436) or 20% (11.1% ± 0.8%; *p* = 0.865) during pressure-regulated volume control ventilation. Inhaled mass fraction was also similar during volume control ventilation with constant inspiratory flow (10.9% ± 0.8%; *p* = 0.869; Fig. 3).

#### Circuit Type and Bends

**Figure 4.**
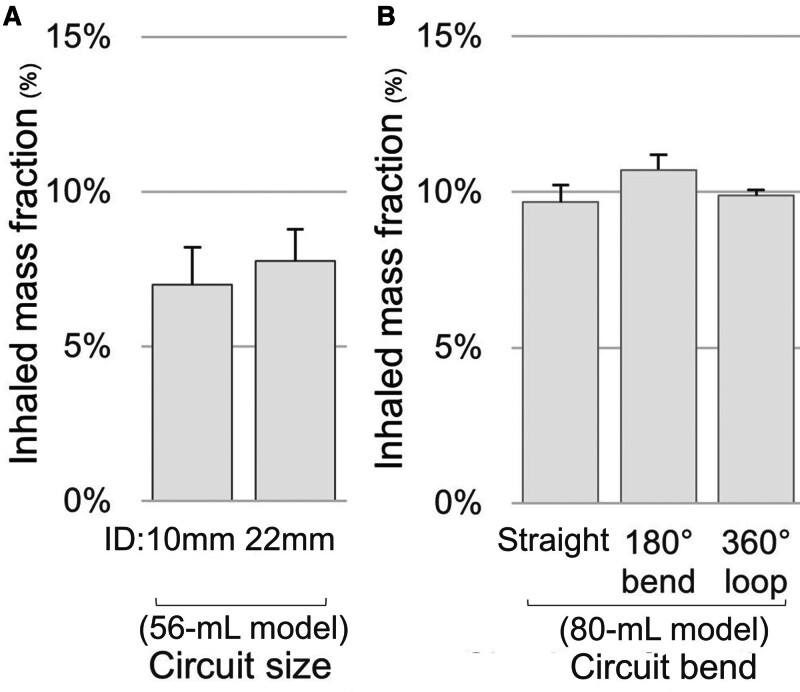
Inhaled mass fraction according to ventilator circuit size and bend. Inhaled mass fraction was measured (**A**) using ventilator circuits with inner diameters (IDs) of 10 and 22 mm in the 56-mL ventilation model and (**B**) with different circuit bends (straight, 180° bend, and 360° loop) in the 80-mL baseline ventilation model. No significant differences were observed among the tested conditions. *Bars* represent the mean values from triplicate experiments, and *error bars* indicate the sd.

In the 56-mL model, inhaled mass fraction did not differ between circuits with IDs of 10 and 22 mm (7.0% ± 1.2% vs. 7.7% ± 1.0%; *p* = 0.293). In the 80-mL baseline model, inhaled mass fraction did not differ significantly when the circuit was modified with either an 180° bend (30-cm diameter; 10.7% ± 0.5%; *p* = 0.067) or a 360° loop (30-cm diameter; 9.9% ± 0.2%; *p* = 0.571), compared with a straight circuit (**Fig. 4**).

#### VMN Position and Drug Dilution

**Figure 5.**
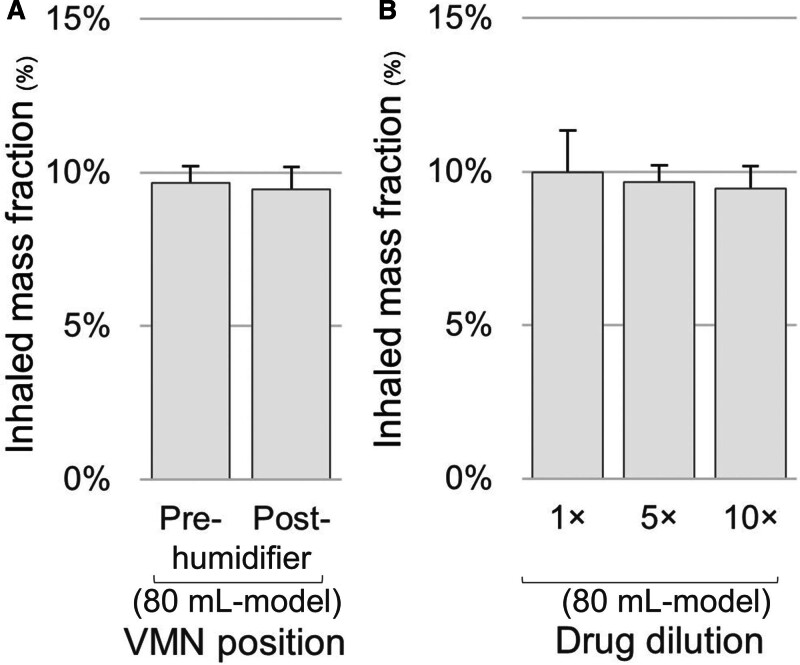
Inhaled mass fraction according to vibrating mesh nebulizer (VMN) position and drug dilution. Inhaled mass fraction was measured (**A**) with the nebulizer positioned pre-humidifier or post-humidifier and (**B**) with different drug dilutions (undiluted [1×], five-fold dilution [5×], and 10-fold dilution [10×]) in the 80-mL baseline ventilation model. No significant differences were observed among the tested conditions. *Bars* represent the mean values from triplicate experiments, and *error bars* indicate the sd.

Inhaled mass fraction did not differ between the VMN position pre- and post-humidifier (9.7% ± 0.5% vs. 9.5% ± 0.7%; *p* = 0.712). In the 80-mL baseline model, inhaled mass fraction did not differ with undiluted salbutamol (10.0% ± 1.4%; *p* = 0.731) and with a 10-fold dilution (9.5% ± 0.7%; *p* = 0.710), compared with the five-fold-diluted condition (**Fig. 5**).

## DISCUSSION

This in vitro study demonstrated that aerosol delivery with a VMN during pediatric invasive ventilation is primarily affected by tidal volume-based ventilation models, with larger tidal volumes being associated with higher inhaled mass fractions. In contrast, variations in other ventilator settings, endotracheal tube size, circuit modifications, VMN position relative to the humidifier, and drug dilution had a minimal impact on aerosol delivery. To our knowledge, this is the first studies to systematically evaluate VMN aerosol delivery across pediatric invasive ventilation models representing a broad range of age groups, while also assessing commonly modified ventilator- and circuit-related conditions in a controlled experimental setting.

A few studies have examined inhaled mass fractions across different tidal volumes in pediatric invasive ventilation models using a VMN. One in vitro study reported inhaled mass fractions of 13.6% and 23.8% in 100- and 500-mL models, respectively (3). These values are consistent with our findings in the 80- and 400-mL ventilation models. Additionally, in our experiments, neither endotracheal tube size nor circuit size significantly affected inhaled mass fraction when each parameter was varied individually. Taken together, these findings suggest that tidal volume is the dominant determinant of inhaled mass fraction during pediatric invasive ventilation. However, since the respiratory rate and inspiratory time varied across the tidal volume-based models to reflect clinically realistic, age-representative ventilation settings, we cannot completely exclude the possibility that these covarying parameters may have contributed to the observed differences in aerosol delivery.

From an aerosol physics perspective, variability in aerosol delivery may be caused by several mechanisms, including gravitational sedimentation within the ventilator circuit, inertial impaction and turbulence-related deposition in narrow passages such as the endotracheal tube, and hygroscopic growth of aerosol particles over time under humidified conditions (9, 10, 13). Although the relative contribution of each mechanism remains uncertain, it is likely that they are all influenced by age- and size-related parameters. Accordingly, we used age-representative pediatric ventilation models with clinically plausible tidal volumes, endotracheal tube sizes, and circuit sizes in order to simulate real-world pediatric invasive ventilation more accurately. In our study, age-related factors, particularly tidal volume, were the dominant determinants of inhaled mass fraction, presumably due to their combined effects on inspiratory flow, Reynolds number, and inertial impaction within ventilation models (9, 14).

In our experiments, neither the bias flow rate nor the inspiratory rise time or ventilator mode significantly affected inhaled mass fraction. Previous studies have reported a higher inhaled mass fraction with a lower bias flow rate (3). The lack of a significant effect in our study may be due to the relatively small difference in the tested bias flow settings (2.0 vs. 3.5 L/min), compared with the larger differences evaluated in previous studies (3). Similarly, inspiratory rise time and ventilator mode did not influence inhaled mass fraction in our experiments. A shorter rise time produces a faster increase in inspiratory flow and a higher peak inspiratory flow, whereas a longer rise time and a constant inspiratory flow during volume-controlled ventilation typically result in a lower peak inspiratory flow (15). In theory, a higher peak inspiratory flow could increase aerosol deposition through turbulence and inertial impaction within the endotracheal tube and along the ventilator circuit (9). However, within the range of settings tested in our experiments, these effects were not evident, and inhaled mass fraction remained unchanged. Further studies using aerodynamic approaches to visualize the behavior of nebulized particles under different inspiratory flow profiles could help clarify these mechanisms.

In our experiments, neither the size nor the bend of the circuit affected inhaled mass fraction. To our knowledge, no previous studies have specifically examined the effect of such modifications to the circuit in pediatric ventilation models. The selection of circuit size may vary in borderline pediatric age groups due to an absence of clear manufacturer recommendations. Additionally, it is common in clinical practice for circuits to sag due to gravity. Our observation of consistent aerosol delivery, despite variations in circuit size and bend, is clinically relevant.

In our experiments, neither the VMN position nor the drug dilution affected the inhaled mass fraction. Previous studies have reported higher delivery when VMNs were positioned on the dry side of the humidifier rather than near the Y-piece, due to the nebulized particle reservoir effect (3–5). The consensus among adults is to position the VMN close to the ventilator and away from the Y-piece (16). Thus, we only simulated the pre- and post-humidifier positions, which showed no significant difference. Regarding drug dilution, the evidence is contradictory: greater dilution results in higher delivery in adult invasive ventilation models with jet nebulizers (6); yet, the effect was minimal in an adult noninvasive ventilation models using VMNs (17). However, to our knowledge, this has not been examined in pediatric invasive ventilation models. In our study, diluting salbutamol beyond the standard five-fold dilution did not provide any additional benefit. Undiluted salbutamol produced similar inhaled mass fractions. This suggests that dilution may be unnecessary for VMN nebulization, given the minimal residual drug dose in the nebulizer compared with jet nebulizers (18). However, the clinical implications of faster drug delivery and the potential risks of adverse effects warrant further research.

This study has limitations. First, as it is an in vitro study, it cannot fully replicate in vivo lung mechanics, regional deposition, or clinical responses. However, we attempted to approximate a clinically plausible pediatric respiratory system by using ventilator settings, circuits, and endotracheal tube configurations based on tidal volume to represent different age groups. Second, since we quantified the amount of inhaled drug captured on the filter before the test lung, our outcome does not directly reflect deposition in the distal airways (i.e., the therapeutic target sites). However, as our primary objective was to compare delivery efficiency across different experimental settings, we believe that this approach appropriately achieved the study’s aim.

## CONCLUSIONS

VMN aerosol delivery increased across tidal volume-based ventilation models and was minimally affected by other ventilator settings, endotracheal tube size, and circuit-related modifications. Consistent aerosol delivery at a given tidal volume suggests that tidal volume is the dominant determinant of aerosol delivery efficacy during pediatric invasive ventilation.

## ACKNOWLEDGMENTS

We express our gratitude to Jun Sakakibara (Meiji University, Kawasaki, Japan) for his valuable insights into interpreting the experimental findings from aerosol and aerodynamic perspectives. We also thank the clinical engineers at St. Marianna University and the laboratory technicians, Ms. Mieko Akita and Ms. Noriko Yamagishi, in the Department of Pediatrics at St. Marianna University School of Medicine, for their assistance in setting up the experimental environment for mechanical ventilation.
